# Supplementary material for: A Critical View of the Application of the APEX Software (Aqueous Photochemistry of Environmentally-Occurring Xenobiotics) to Predict Photoreaction Kinetics in Surface Freshwaters
Source: Molecules. 2019 Dec 18;25(1):9. doi: 10.3390/molecules25010009 (PMC7017383; doi:10.3390/molecules25010009)
Supplement: Supplementary file 1 [file molecules-25-00009-s001.zip › 3.2.4_gcc-4.4.0.zip!/3.2.4_gcc-4.4.0/3.2.4_gcc-4.4.0/doc/octave/html/liboctave/Nonlinear-Functions.html]

Nonlinear Functions - Octave C++ Classes


Next: Nonlinear Equations,
Previous: Ranges,
Up: Top


---

## 7 Nonlinear Functions

— : **NLFunc** (void)  
— : **NLFunc** (const nonlinear\_fcn)  
— : **NLFunc** (const nonlinear\_fcn, const jacobian\_fcn)  
— : **NLFunc** (const NLFunc &a)

— : NLFunc& **operator =** (const NLFunc &a)

— : nonlinear\_fcn **function** (void) const;

— : NLFunc& **set\_function** (const nonlinear\_fcn f)

— : jacobian\_fcn **jacobian\_function** (void) const;

— : NLFunc& **set\_jacobian\_function** (const jacobian\_fcn j)
